# The Adaptive Cellular State Model: A Conceptual Framework Linking Dietary Quality to Exercise-Induced Cellular Adaptation

**DOI:** 10.3390/cells15181629

**Published:** 2026-09-08

**Authors:** Mario Muñoz-López, Gonzalo Quesada-Fernández, Edgar Simón Sancho-Haro, José Francisco López-Gil, Miguel López-Moreno, José Francisco Tornero-Aguilera

**Affiliations:** 1 Department of Sport Sciences, Faculty of Sport and Health Sciences, Fit Generation Research Institute, AD500 Andorra la Vella, Andorra; 2 Department of Nutrition and Dietetics, Faculty of Sport and Health Sciences, Fit Generation Research Institute, AD500 Andorra la Vella, Andorra; 3 School of Medicine, Universidad Espíritu Santo, Samborondón 092301, Ecuador; 4 Faculty of Health Sciences, Universidad Autónoma de Chile, Temuco 4780000, Chile; 5 Institute of Health and Sport Sciences, Faculty of Health Sciences, Universidad Francisco de Vitoria, 28223 Madrid, Spain

**Keywords:** adaptive cellular state, exercise adaptation, dietary quality, cellular plasticity, mitochondrial biogenesis, immunometabolism, metabolic flexibility, molecular nutrition

## Abstract

**Highlights:**

**What are the main findings?**

- Exercise provides the mechanical, energetic, redox and immune–endocrine perturbations that initiate cellular adaptation, whereas the internally generated perturbation and its subsequent translation into durable remodelling may vary according to the biological context in which exercise occurs.
- The Adaptive Cellular State (ACS) is proposed as a pre-existing, dynamic and multidimensional biological configuration that may condition how exercise-induced perturbations are received, decoded, executed and retained across repeated adaptive cycles. The model organizes nine interacting domains and treats ACS as a prospective latent construct rather than as a validated biomarker, clinical category or outcome inferred retrospectively from successful adaptation.

**What are the implications of the main findings?**

- Habitual dietary quality is proposed as one potential modifier of selected ACS-related domains, with nutrient adequacy, food processing, food matrix and the foods displaced by alternative choices considered more informative than vegan, vegetarian or omnivorous labels alone. Current evidence does not establish that any dietary pattern produces a superior ACS profile or greater training adaptation.
- The ACS Model generates prospective and falsifiable hypotheses that require baseline assessment of candidate ACS domains, independent measurement of subsequent adaptation and demonstration of incremental information beyond conventional cardiometabolic, fitness and training-history variables before any predictive or clinical application can be considered.

**Abstract:**

Exercise is a recurrent multidimensional perturbation that initiates biological adaptation, yet responses to comparable training programmes remain heterogeneous. This critical narrative Review introduces the Adaptive Cellular State (ACS) Model as a heuristic framework for examining how the biological context present before training may contribute to variation in the subsequent processing and consolidation of exercise-induced perturbations. ACS is defined as a pre-existing, dynamic and multidimensional biological configuration that may condition how exercise-induced perturbations are received, decoded, executed and retained across repeated adaptive cycles. The model organizes nine interacting domains—mitochondrial quality, metabolic flexibility, nutrient sensing, redox responsiveness, inflammatory tone, microbiota-derived signalling, endothelial function, proteostasis and epigenetic readiness—and proposes four emergent properties: gain, fidelity, biological noise and memory. These domains and properties are theoretical constructs rather than validated ACS measurements or clinical categories. Exercise remains the indispensable initiating stimulus, whereas nutrition may act through distinct routes: acute nutritional conditions may modify the internal perturbation generated during exercise, habitual dietary quality may influence selected components of the pre-existing biological context, and recovery nutrition may provide substrates or modify conditions relevant to remodelling. Nutrient-adequate dietary patterns emphasizing minimally processed foods and a diverse plant-food component represent plausible candidate exposures, but current evidence does not establish superiority over appropriately constructed high-quality omnivorous patterns or demonstrate greater long-term training adaptation. The ACS Model should therefore be interpreted as a prospective and falsifiable research framework whose validity depends on whether prespecified baseline ACS-related measures provide reproducible information about durable adaptation beyond conventional metabolic, fitness and training-history variables.

## 1. Introduction

Exercise represents a recurrent biological perturbation rather than a simple behavioural exposure. Each contractile bout forces cells to restore homeostasis under acute energetic, mechanical, redox and inflammatory stress [1,2]. This process transiently alters adenosine triphosphate turnover, intracellular calcium flux, reactive oxygen species (ROS) production and vascular shear stress [3]. Repeated exposure to these homeostatic disruptions activates coordinated transcriptional and translational programmes that progressively support mitochondrial remodelling, metabolic regulation, proteostasis and systemic adaptation [1,2,3,4]. Consequently, durable training adaptations reflect not only the initiating exercise perturbation but also the biological context in which exercise-induced signals are processed and translated into structural and functional remodelling.

This adaptive capacity exhibits profound interindividual variability. Individuals exposed to comparable prescribed exercise programmes frequently develop markedly different physiological responses [5]. Improvements in cardiorespiratory fitness, insulin sensitivity, mitochondrial oxidative capacity and lean mass vary substantially even when the external training dose is closely standardized. Improvements in cardiorespiratory fitness, insulin sensitivity, mitochondrial oxidative capacity and lean mass vary substantially even when the training dose is rigidly standardized. Contemporary exercise medicine increasingly recognizes this heterogeneity as a fundamental biological reality rather than statistical noise around an average treatment effect [5]. While genetic architecture, chronological age, sex and baseline fitness contribute to this variance, these static factors do not fully explain why some individuals display robust adaptive remodelling while others show attenuated responses to the same contractile stimulus. This observation highlights a central mechanistic problem: comparable prescribed training does not necessarily produce equivalent internal perturbations or equivalent biological responses.

Extensive mechanistic research has characterized the signalling networks through which muscle contraction initiates adaptation, including Nrf2-, AMPK-, CaMK- and mTORC1-related pathways involved in mitochondrial remodelling, autophagy, protein turnover and redox regulation [2,3,6,7,8]. These signals, however, operate within a pre-existing biological environment shaped by proteostatic, metabolic, microbial and cardiometabolic processes [6,8,9,10,11,12,13]. Although nutrition can influence many of these processes, existing models do not explicitly organize them as an adaptation-specific biological context through which exercise-induced signals are generated, processed and consolidated. This conceptual gap motivates the present Review.

Among the potentially modifiable determinants of this biological environment, habitual diet is particularly relevant because its influence extends beyond individual exercise bouts. Dietary exposures can affect microbial and metabolite signalling [9,10,11], cardiometabolic and vascular physiology [12,13], and energy intake and body-weight regulation through differences in food processing [14,15,16]. Accordingly, the principal exposure considered in this Review is dietary quality, defined broadly by nutrient adequacy, degree of processing, food matrix, overall dietary composition and the foods displaced by alternative choices [12,13,14,15,16,17], rather than by vegan, vegetarian or omnivorous labels alone.

We refer to this pre-existing biological context as the Adaptive Cellular State (ACS), defined here as the dynamic and multidimensional configuration of cells and tissues that conditions how exercise-induced perturbations are received, decoded, executed and retained across repeated adaptive cycles. ACS is proposed as a latent, adaptation-specific construct rather than a synonym for metabolic health, metabolic flexibility, hormetic responsiveness, short-term exercise readiness or the eventual training response itself. Critically, ACS must be characterized prospectively, before the adaptation it is intended to explain or predict; inferring ACS retrospectively from the magnitude of the resulting adaptation would constitute circular reasoning.

Under this formulation, exercise remains the primary and indispensable adaptive stimulus: diet cannot reproduce the multidimensional mechanical, energetic and biochemical information generated by muscle contraction [1,2,3]. Dietary exposures may instead act through two conceptually distinct routes: acute or proximal nutritional conditions can modify the internal perturbation generated during exercise, whereas habitual diet may shape the pre-existing and recovery environment in which that perturbation is processed [9,10,11,12,13,17], including energy- and nutrient-sensing pathways and metabolic flexibility [18,19,20].

The Adaptive Cellular State Model organizes this interaction across nine interconnected domains: mitochondrial quality, metabolic flexibility, nutrient sensing, redox responsiveness, inflammatory tone, microbiota-derived signalling, endothelial function, proteostasis and epigenetic readiness. These domains are not proposed as novel biological processes, nor as components that must vary concordantly. Their proposed novelty lies in treating their pre-exercise configuration as a multidimensional, prospectively testable moderator of the relationship between exercise perturbation and subsequent adaptation.

The contribution of this Review is therefore integrative rather than the proposal of new molecular pathways. We examine how established exercise- and diet-responsive processes can be organized within an adaptation-specific framework, describe the proposed behaviour of ACS through the theoretical properties of gain, fidelity, biological noise and memory, and define predictions that can be prospectively supported, refined or refuted. The model is presented as a heuristic and testable research framework, not as a validated biomarker, predictive tool or established explanation of interindividual training responsiveness.

## 2. Review Approach

This work was conducted as a critical narrative review aimed at developing and critically evaluating a mechanistic framework linking habitual dietary quality to exercise-induced cellular adaptation. Evidence was considered across three interconnected domains: (i) molecular and cellular mechanisms involved in exercise-induced signalling and remodelling; (ii) effects of habitual dietary exposures on biological systems relevant to adaptation; and (iii) human evidence examining relationships among diet, exercise and adaptive outcomes. The purpose was conceptual integration and critical appraisal rather than exhaustive evidence retrieval or quantitative effect estimation.

Literature identification combined targeted database searching with backward and forward citation tracking of relevant primary studies and evidence syntheses. Searches were organized around exercise adaptation, dietary quality and the biological domains represented in the proposed framework. Because this was a narrative rather than a systematic review, the literature identification was purposive and iterative rather than designed to provide exhaustive retrieval or a reproducible study census. Human intervention and longitudinal studies were given greatest weight when they directly informed exercise adaptation or dietary-pattern effects, followed by observational human evidence where appropriate. Cellular and animal studies were used to clarify mechanisms that cannot readily be interrogated in humans and were treated as indirect evidence for human exercise adaptation. Similarly, studies of acute substrate manipulation, isolated nutrients or bioactive compounds were used to interrogate specific pathways, not as evidence that habitual whole-diet patterns produce the same effects. Sources were selected according to conceptual relevance, methodological robustness, directness to the question under consideration and their capacity to support, qualify or challenge the proposed framework. Primary research and controlled human evidence were prioritized where available, while seminal mechanistic studies and high-quality evidence syntheses were used to establish biological context. Null findings, contradictory observations and plausible alternative explanations were deliberately considered alongside supportive evidence. Nevertheless, because study selection and evidential weighting necessarily involved author judgement, confirmation bias and selective emphasis cannot be excluded. No formal systematic screening, duplicate study selection, risk-of-bias assessment or certainty grading was performed. Evidence was synthesized narratively, with explicit attention to study design, biological directness and translational limitations. Accordingly, the coherence of the evidence assembled here should not be interpreted as equivalent to systematic evidence certainty, and the ACS Model is evaluated as a heuristic framework whose propositions require prospective experimental testing.

## 3. Cellular Adaptation to Exercise: A Mechanistic Overview

Exercise simultaneously perturbs cellular energetics, mechanical loading, calcium homeostasis, redox state, oxygen delivery and inter-organ signalling [1,2,3,4]. These perturbations are sensed by overlapping regulatory networks that coordinate energy restoration, transcriptional responses, protein turnover and organelle remodelling [3,7,8]. With repeated exposure and recovery, these transient responses contribute to more durable changes in oxidative metabolism, proteostasis and tissue function [1,4]. The purpose of this section is therefore not to review each canonical pathway in detail, but to identify the principal processes through which an acute exercise perturbation is converted into cellular and tissue remodelling.

### 3.1. Mechanical and Metabolic Signalling

Muscle contraction rapidly alters ATP turnover, intracellular calcium, redox state and local oxygen and substrate demand [2,3,18]. AMPK responds to energetic stress by coordinating substrate use, energy restoration and pathways involved in mitochondrial and autophagic remodelling [7,8,18], while metabolic flexibility influences the capacity to redistribute substrates as energetic demands change [19]. In parallel, mTORC1 integrates mechanical input, amino acid availability, growth-factor signalling and cellular energy status to regulate translational activity and structural remodelling [20].

AMPK- and mTORC1-related signalling should not be interpreted as a simple catabolic–anabolic switch. Their relative activity varies with the exercise stimulus, nutrient availability and temporal context, allowing energetic restoration, quality control and biosynthesis to coexist across the adaptive response [1,2,3].

**Figure 1:**
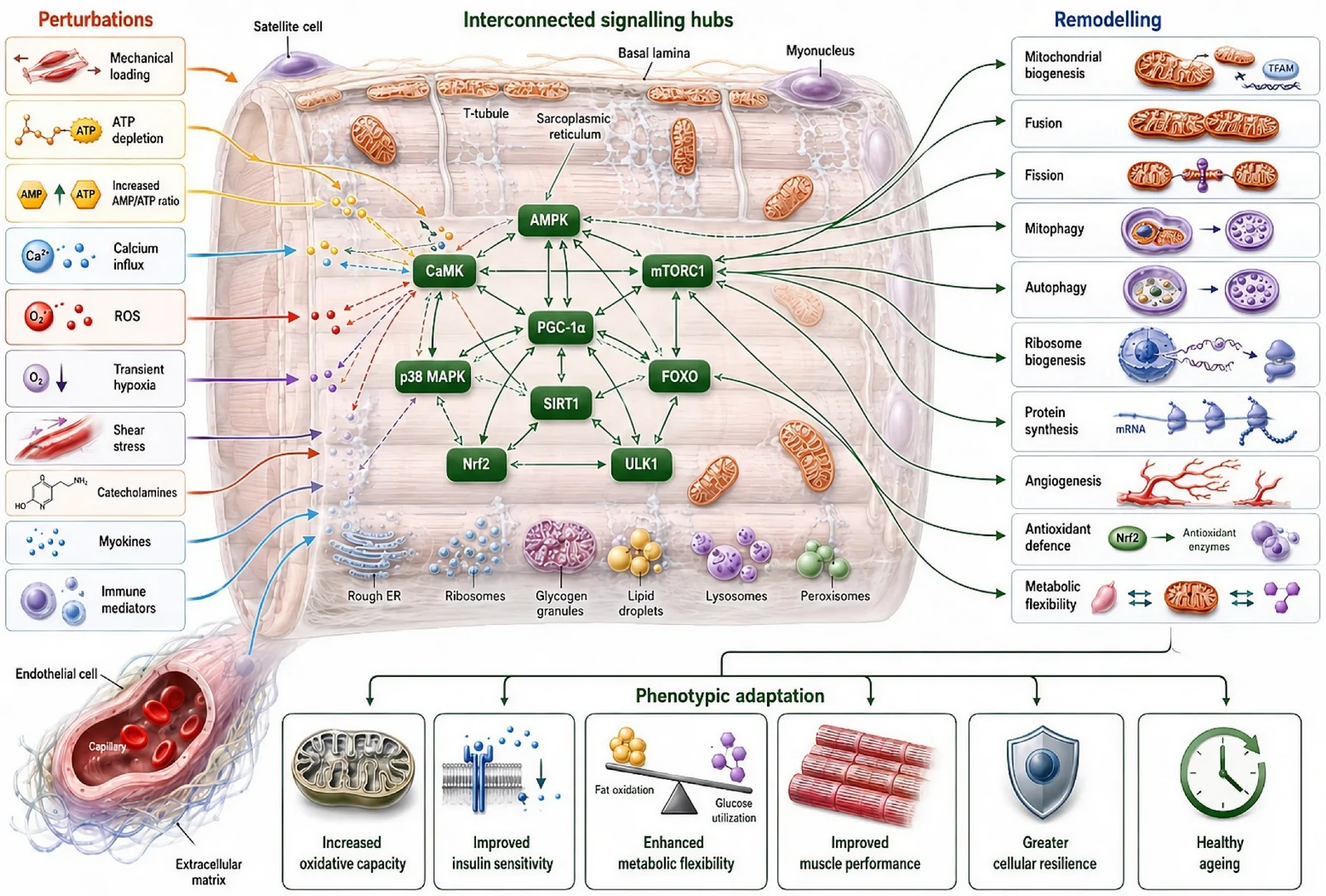
Exercise as a multidimensional cellular perturbation. Mechanical, energetic, calcium-dependent, redox, vascular and immune–endocrine inputs converge on overlapping signalling networks that coordinate mitochondrial, proteostatic, translational and vascular remodelling. Connections are schematic and represent context-dependent interactions rather than fixed one-to-one causal relationships. Solid arrows denote principal directional relationships, whereas dotted arrows denote context-dependent crosstalk or regulatory interactions rather than fixed linear causal pathways.

Calcium-dependent signalling provides another route through which contractile activity is coupled to transcriptional remodelling. CaMK- and calcineurin-related pathways contribute to exercise-responsive transcription [21], while p38 MAPK, AMPK and related stress-sensitive signals converge on PGC-1α-associated mitochondrial programmes [3,7]. SIRT1 and FOXO provide additional energy- and stress-responsive regulatory inputs [7]. Mitochondrial adaptation therefore arises from converging signals rather than from activation of a single linear pathway. Exercise-induced ROS are likewise context-dependent signals rather than exclusively markers of cellular damage [6,22]. Mechanistic evidence, including preclinical work, supports Nrf2-mediated regulation of endogenous antioxidant and mitochondrial responses [22,23], while human intervention studies indicate that high-dose antioxidant supplementation can attenuate selected exercise adaptations [24]. Redox regulation should therefore be considered non-linear: the biological consequence depends on the magnitude, timing and context of the signal rather than on maximal suppression of oxidative activity [22,23,24]. Together, energetic, mechanical, calcium-dependent and redox signals provide overlapping inputs to exercise-induced remodelling [2]. Their importance lies not in the isolated activation of individual pathways, but in whether transient signalling is subsequently translated into durable structural and functional change (Figure 1).

### 3.2. Mitochondrial Remodelling, Autophagy and Ribosome Biogenesis

Transient signalling acquires functional relevance only when it is translated into structural remodelling. Mitochondrial adaptation involves not only changes in mitochondrial content but also coordinated regulation of biogenesis, fusion, fission, cristae organization and selective removal of dysfunctional organelles [25,26]. PGC-1α integrates several exercise-responsive signals involved in this remodelling [3,7], whereas mitophagy and broader autophagic processes contribute to organelle quality control and recycling of damaged cellular components [8,18,25].

These degradative and biosynthetic processes operate coordinately rather than as opposing programmes. Efficient remodelling requires removal of dysfunctional proteins and organelles alongside synthesis of new cellular components [8,25]. The relevant adaptive outcome is therefore the quality and function of the resulting cellular architecture, not simply the accumulation of mitochondrial or protein mass.

Structural remodelling also depends on translational capacity. Ribosome biogenesis expands the cellular machinery available for protein synthesis [27], and interindividual differences in this response may contribute to variability in hypertrophic adaptation [28]. Translational capacity can therefore constrain how effectively exercise-induced anabolic signalling is converted into new cellular structure [27,28]. Endurance and resistance exercise differ in their dominant phenotypic outcomes but share several elements of mitochondrial, proteostatic and translational remodelling [1,2,3,26,27]. The magnitude and temporal organization of these processes vary with the training stimulus, but the central point for the present framework is common to both modalities: acute pathway activation is insufficient unless it is successfully executed through organelle turnover, protein renewal and structural remodelling. This distinction between signalling and execution becomes central to the model developed below.

### 3.3. Inflammatory, Redox and Epigenetic Regulation

Exercise also produces transient immune and redox responses that participate in metabolic regulation, tissue repair and subsequent remodelling [22,29,30]. These responses should be distinguished from the persistent low-grade inflammatory activity characteristic of ageing, obesity and metabolic disease [31,32]. The relevant distinction is therefore not simply whether inflammatory signalling is present, but its source, magnitude, timing and resolution [29,30,31,32]. Skeletal muscle contributes to this systemic response through contraction-induced myokine signalling [4]. Interleukin-6 illustrates the importance of biological context: when released during exercise, it participates in metabolic regulation and in the subsequent induction of anti-inflammatory mediators [29,33]. The physiological meaning of an inflammatory signal therefore depends strongly on its cellular source and temporal pattern rather than on the identity of the mediator alone. Redox signalling follows a similar context-dependent pattern. Transient exercise-induced ROS participate in redox-sensitive transcription and endogenous antioxidant regulation [6,22,23], whereas excessive or poorly timed suppression of these signals can attenuate selected adaptive responses [24]. Importantly, several mechanistic components of this pathway are supported substantially by cellular or animal evidence and should not be interpreted as direct demonstrations of durable human training adaptation. Inflammatory and redox processes also interact with mitochondrial metabolism, immune-cell recruitment and tissue remodelling [30,34]. These interactions extend beyond skeletal muscle and contribute to the systemic nature of exercise adaptation [4]. For the ACS framework, their importance lies in the distinction between transient, appropriately resolved signalling and a chronically altered background state that may modify how subsequent exercise perturbations are processed. Repeated exercise can also produce persistent changes in DNA methylation, histone regulation and non-coding RNA expression [35]. Such changes provide a plausible molecular basis for history-dependent responsiveness to subsequent exercise, although the duration, tissue specificity and functional importance of this putative epigenetic memory remain incompletely defined in humans [35]. Epigenetic regulation should therefore be treated as one potential contributor to adaptive history rather than as evidence of a fixed or directly measurable memory state. Inflammatory, redox and epigenetic processes therefore represent interacting components of the biological context in which repeated exercise responses are generated and consolidated [1,29,35]. Their relevance to the ACS Model lies in their potential to condition adaptation, not in their use as interchangeable or individually sufficient markers of adaptive competence.

## 4. Dietary Modulation of the Biological Context for Exercise Adaptation

The preceding section established that exercise provides the initiating mechanical, energetic, calcium-dependent, redox and immune–endocrine perturbations. Habitual diet may modify the biological context in which these perturbations are subsequently processed, but it is not proposed to reproduce the exercise stimulus itself. Throughout this section, evidence from acute substrate manipulation, isolated nutrients or individual metabolites is used only to interrogate specific mechanisms and is not treated as evidence that a habitual whole-diet pattern produces the same effect. We therefore distinguish the longer-term influence of habitual diet on the pre-existing ACS from acute nutritional conditions that may modify the internal perturbation generated during a particular exercise bout.

### 4.1. Energy Availability and Nutrient Sensing

Nutrient-sensing pathways engaged by exercise are also responsive to the nutritional environment between exercise bouts [18,20,36]. AMPK, mTORC1 and sirtuin-related signalling integrate information about cellular energy status, amino acid availability and redox state, providing a mechanistic basis through which habitual nutritional exposure may influence the pre-exercise biological context [18,20,36]. However, the magnitude and direction of this influence cannot be inferred from the resting activity of any single pathway, and chronic nutrient availability should not be interpreted as a simple determinant of subsequent exercise responsiveness. Metabolic flexibility illustrates this distinction at the physiological level. Defined as the capacity to adjust fuel oxidation in response to changes in supply and demand [19], it is both responsive to habitual metabolic conditions and relevant to substrate handling during exercise. Sustained energy surplus and lipid oversupply can impair insulin signalling and substrate switching [19], but metabolic flexibility remains only one component of the proposed ACS and should not be used as a surrogate for adaptive competence as a whole. Chronic nutrient excess and dietary restriction can also modify nutrient-sensing and quality-control pathways [20,37]. Dietary-restriction research demonstrates coordinated changes in AKT–FOXO, mTOR, AMPK and NAD^+^-related signalling, with much of the mechanistic evidence derived from non-human models [37]. These findings establish biological overlap between dietary regulation and pathways involved in exercise adaptation, but they do not demonstrate that dietary restriction, chronic mTORC1 suppression or any particular resting signalling configuration improves training adaptation in humans. NAD^+^ metabolism provides a related mechanistic example because sirtuin activity depends on NAD^+^ availability and links cellular energy status to mitochondrial regulation [36]. Dietary or supplemental manipulation of this pathway can therefore engage regulatory chemistry also used during exercise adaptation, but current evidence does not establish that modifying NAD^+^ availability enhances long-term training adaptation in humans [36]. Acute carbohydrate availability illustrates a different nutritional pathway. Performing selected exercise sessions with reduced glycogen can modify AMPK-, p38 MAPK- and PGC-1α-related signalling compared with more carbohydrate-replete conditions [38]. This demonstrates that nutritional conditions immediately surrounding exercise can alter the internal molecular perturbation generated by a prescribed workload. It should not, however, be interpreted as evidence about habitual dietary quality or ACS, and amplified acute signalling has not consistently translated into superior functional outcomes [38]. Accordingly, habitual nutritional exposure and acute nutrient availability should be treated as related but distinct influences: the former may contribute to the pre-existing multidimensional ACS, whereas the latter may alter the internal perturbation generated during a particular exercise bout.

### 4.2. Food Matrix, Microbiota and Bioactive Compounds

**Figure 2:**
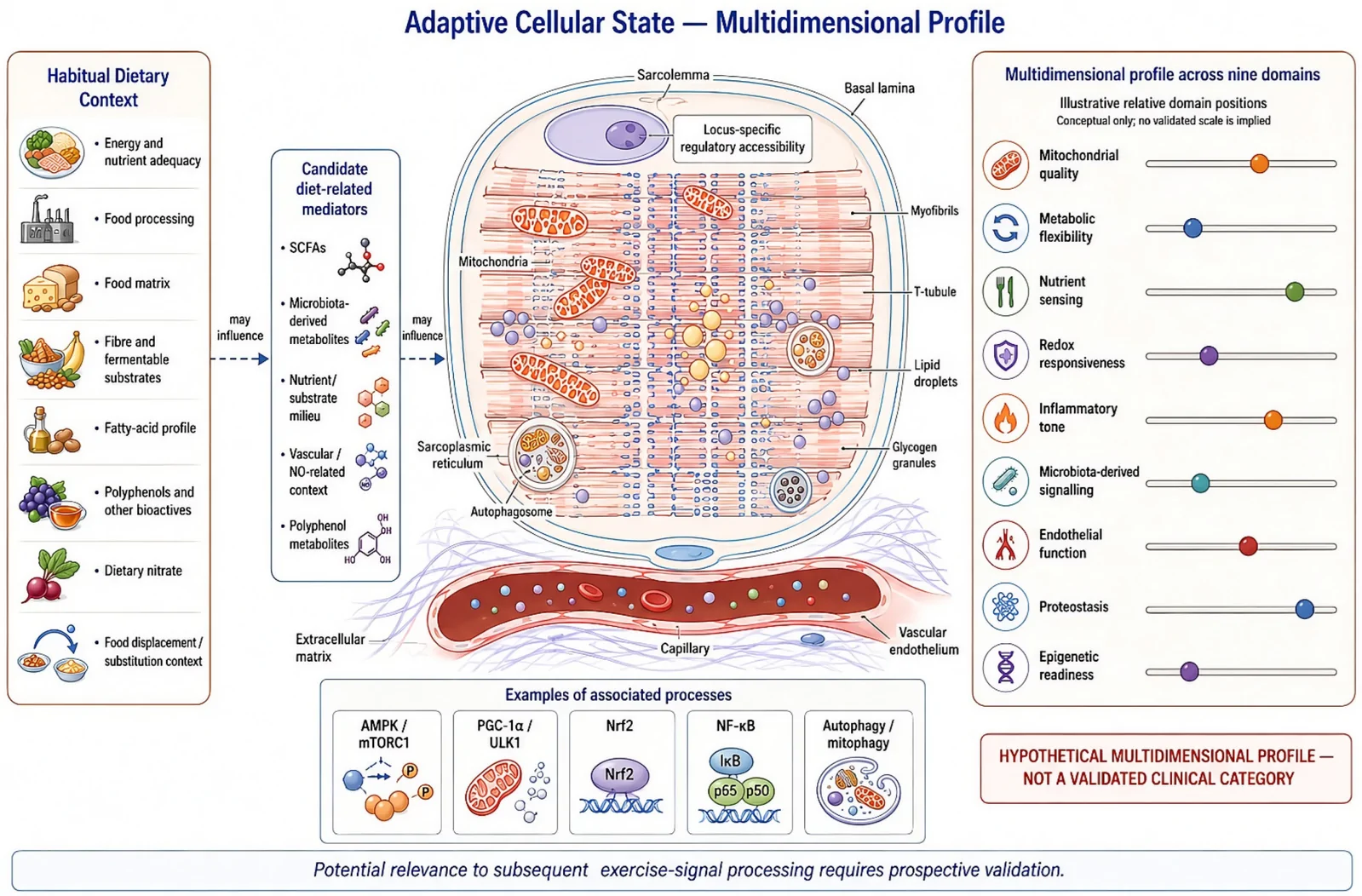
Dietary modulation of the Adaptive Cellular State (ACS) as a hypothetical multidimensional profile. Habitual dietary context may influence candidate diet-related mediators and biological domains represented in the ACS, including mitochondrial quality, metabolic flexibility, nutrient sensing, redox responsiveness, inflammatory tone, microbiota-derived signalling, endothelial function, proteostasis and epigenetic readiness. Domain positions are illustrative only and do not represent validated scales, categories or an overall ACS score. Molecular processes shown are examples of pathways associated with these domains rather than validated ACS biomarkers. The relationships depicted are conceptual and require prospective validation; they should not be interpreted as established effects of any specific dietary pattern on exercise adaptation.

Beyond energy and macronutrient provision, the food matrix influences digestion, bioaccessibility and the substrates reaching the intestinal microbiota [11]. Microbial metabolism can in turn generate circulating compounds capable of interacting with metabolic, immune and vascular pathways [11,39]. Within the present framework, these processes are relevant because they provide plausible routes through which habitual dietary composition could modify selected ACS domains before exercise occurs. Fermentation of dietary fibre generates short-chain fatty acids (SCFAs), which can act as substrates and signalling molecules through G-protein-coupled receptors and histone-deacetylase-related mechanisms [39]. These pathways provide a plausible connection between habitual fibre intake, microbial metabolism and host metabolic or immune regulation [11,39]. Their relevance to ACS remains mechanistic, however: evidence that SCFAs influence these pathways should not be interpreted as evidence that greater fibre intake or SCFA exposure enhances long-term exercise adaptation in humans. Bidirectional exercise–microbiota interactions provide additional mechanistic plausibility. In a preclinical model, exercise-associated lactate was metabolized by gut bacteria into propionate, with subsequent effects on endurance capacity [40]. This finding supports biological communication between host exercise metabolism and the microbiota, but its contribution to long-term human training adaptation remains unknown. The same microbial axis can also generate metabolites with uncertain or potentially adverse vascular relevance. Trimethylamine N-oxide (TMAO), produced from microbial metabolism of precursors including choline and L-carnitine, has been associated with cardiovascular phenotypes [41]. Its causal importance in humans and its relevance to exercise adaptation remain unresolved; it is therefore used here only as an example of a diet–microbiota–vascular pathway, not as a marker of low ACS or an established inhibitor of adaptation. Microbial metabolism of dietary polyphenols provides another example. Ellagitannins can be converted into urolithin A, which activates mitophagy-related pathways in preclinical models [42]. Early human supplementation studies have reported changes in mitochondrial-related molecular biomarkers [43], but these findings do not establish improved mitochondrial function, greater exercise responsiveness or enhanced training adaptation. Urolithin A is therefore used here only as an example of a microbiota-derived metabolite capable of engaging a pathway represented within the ACS framework [42,43]. Collectively, food-matrix and microbiota-derived signals provide biologically plausible routes through which habitual diet may influence metabolic, inflammatory, vascular and mitochondrial domains relevant to the ACS. The evidence is strongest for pathway engagement and host physiology, not for modification of durable exercise adaptation. Figure 2 therefore represents these relationships as hypotheses about the pre-existing biological context rather than as established diet-to-adaptation effects.

### 4.3. Cellular Resilience as a Functional Consequence of ACS

Cellular resilience is treated here as a potential functional consequence of ACS rather than as a synonym for the construct itself. It refers to the capacity of cells and tissues to tolerate perturbation, restore function and undergo remodelling rather than progressive dysfunction [44]. Within the model, resilience may emerge from the combined behaviour of several ACS domains, but it should not be inferred retrospectively from a favourable training response. Chronic inflammatory activity provides one example of how baseline biology could influence resilience. Habitual diet contributes to the metabolic and inflammatory environment [13,31], whereas persistent metaflammation characterizes several ageing- and obesity-related states [31,32]. Within the ACS framework, such background activity is hypothesized to increase the biological noise against which transient exercise-induced inflammatory and redox signals occur. Whether reducing this background activity increases subsequent training adaptation independently of other metabolic changes remains to be demonstrated. Proteostasis provides another candidate pathway. Dietary polyamines such as spermidine have been proposed as caloric-restriction mimetics and can influence autophagic and inflammatory processes [45]. Together with the mitophagy-related effects reported for selected microbial metabolites [42,43], these observations show that nutritional exposures can engage quality-control pathways also recruited during exercise adaptation. They do not establish that these exposures increase ACS or improve long-term training adaptation in humans. Several ACS domains overlap with processes represented in contemporary frameworks of biological ageing, including nutrient sensing, mitochondrial function, proteostasis, autophagy, inflammation and dysbiosis [44]. This overlap provides useful biological context but does not make ACS equivalent to a hallmarks-of-ageing construct. The proposed distinction is functional and prospective: ACS concerns whether a pre-existing multidimensional biological profile contributes information about subsequent exercise adaptation beyond conventional metabolic, fitness or ageing-related measures. Dietary influences on epigenetic regulation and microbial metabolites [35,39,44] may contribute to this profile, but their role in durable human exercise adaptation remains a hypothesis to be tested directly.

## 5. Nutritional Modulation of the Execution Phase of Exercise Adaptation

The biological machinery responsible for sensing exercise-induced stress operates instantaneously, yet the physical consolidation of this signal into functional tissue architecture requires sustained execution [2,3]. Once a muscle contraction has activated its energetic, mechanical and redox cascades, the cell must convert this transient information into durable structures over the subsequent hours and days [3]. We refer to this process as the *execution phase*, during which transient exercise-induced molecular signals are progressively translated into durable structural and functional remodelling. The recovery period therefore provides one biological window during which nutritional conditions may influence the execution of exercise-initiated remodelling [38].

Although exercise remains the indispensable initiating stimulus, nutritional conditions during recovery may provide substrates or modify regulatory environments relevant to mitochondrial, proteostatic, structural and vascular remodelling [38,46]. This execution phase should be distinguished from the pre-existing ACS described in Section 4: the former concerns processes occurring after an exercise perturbation has been generated, whereas the latter refers to the multidimensional biological context present before the adaptive outcome being studied. Evidence from isolated nutrients or acute nutritional manipulations is used here to interrogate these execution mechanisms, not as evidence that habitual dietary quality necessarily enhances long-term training adaptation.

### 5.1. Mitochondrial Function, Metabolic Flexibility and Oxidative Stress

The mitochondrial-biogenic programme initiated by exercise continues during recovery, creating a temporal window in which nutritional conditions may interact with redox-sensitive remodelling [25,26]. High-dose antioxidant supplementation provides an informative example. In Gómez-Cabrera et al., vitamin C supplementation attenuated the improvement in endurance capacity in a small human experiment, whereas suppression of PGC-1α-, NRF-1-, TFAM- and related mitochondrial-biogenesis responses was demonstrated in the accompanying rat model [47]. Subsequent randomized human evidence reported attenuation of selected molecular markers of mitochondrial adaptation with high-dose vitamins C and E [48]. These findings show that nutritional manipulation can interfere with selected redox-sensitive responses, but the molecular and functional evidence should not be conflated across species or interpreted as proof that habitual dietary antioxidant exposure determines training adaptation. This blunting effect exhibits strong dose and chemical-form specificity. Supraphysiological doses of isolated synthetic vitamins differ fundamentally from the modest, matrix-bound polyphenol and micronutrient exposure derived from a whole-food dietary pattern. The defensible mechanistic position remains narrow and highly directional. Chronic, high-dose antioxidant intake timed around training sessions can attenuate selected redox-sensitive molecular or training responses. Conversely, the precise modulatory effect of ordinary dietary antioxidant quality on this pathway remains uncertain and should not be overstated.

Redox regulation and recovery substrate availability represent distinct potential constraints on mitochondrial remodelling. Exercise adaptation relies upon repeated bouts demanding continuous intracellular fuel redistribution [19]. Nutritional strategies that deliberately prolong low-glycogen or low-energy availability may amplify selected acute molecular responses [38]. However, these responses do not consistently translate into superior functional or long-term training adaptation and may compromise training quality. Diet therefore does not create the mitochondrial signal, but may modify the redox and substrate context in which that signal is transcribed, translated and consolidated.

### 5.2. Anabolic Signalling, Muscle Remodelling and Recovery

The anabolic arm of adaptation illustrates this identical execution-phase principle through a different biological currency. Resistance exercise primes mTORC1 and markedly sensitizes the cellular translational apparatus [20]. However, this mechanical priming is insufficient in isolation. Net protein accretion requires adequate amino acid availability during recovery, although the optimal amount, distribution and timing depend on the individual and training context. Crucially, muscle protein synthesis after a contractile bout is highly transient and inherently saturable. It rises rapidly, peaks, and returns toward baseline within a few hours even when circulating amino acids and intracellular anabolic signalling remain highly elevated [49]. This physiological refractoriness defines the classical “muscle-full” effect [46]. Together, these observations suggest that the execution phase represents a biological bottleneck between exercise-induced signalling and durable structural adaptation [3,27,28].

Nutritional factors may influence the efficiency with which this bottleneck is traversed by supporting amino acid availability and nutrient-sensitive anabolic signalling [20,46], although they cannot override the intrinsic biological limits governing protein accretion [46,49]. The decoupling of sustained molecular signalling from finite protein synthesis represents the central execution-phase insight [20,46,49]. Exercise transiently increases anabolic sensitivity, and dietary amino acid provision contributes to the extent to which this exercise-induced opportunity is translated into protein accretion [3,46]. Nevertheless, total conversion remains bounded by the intrinsic biology and translational capacity of the myofibre rather than by substrate supply alone [27,28,46,49].

**Figure 3:**
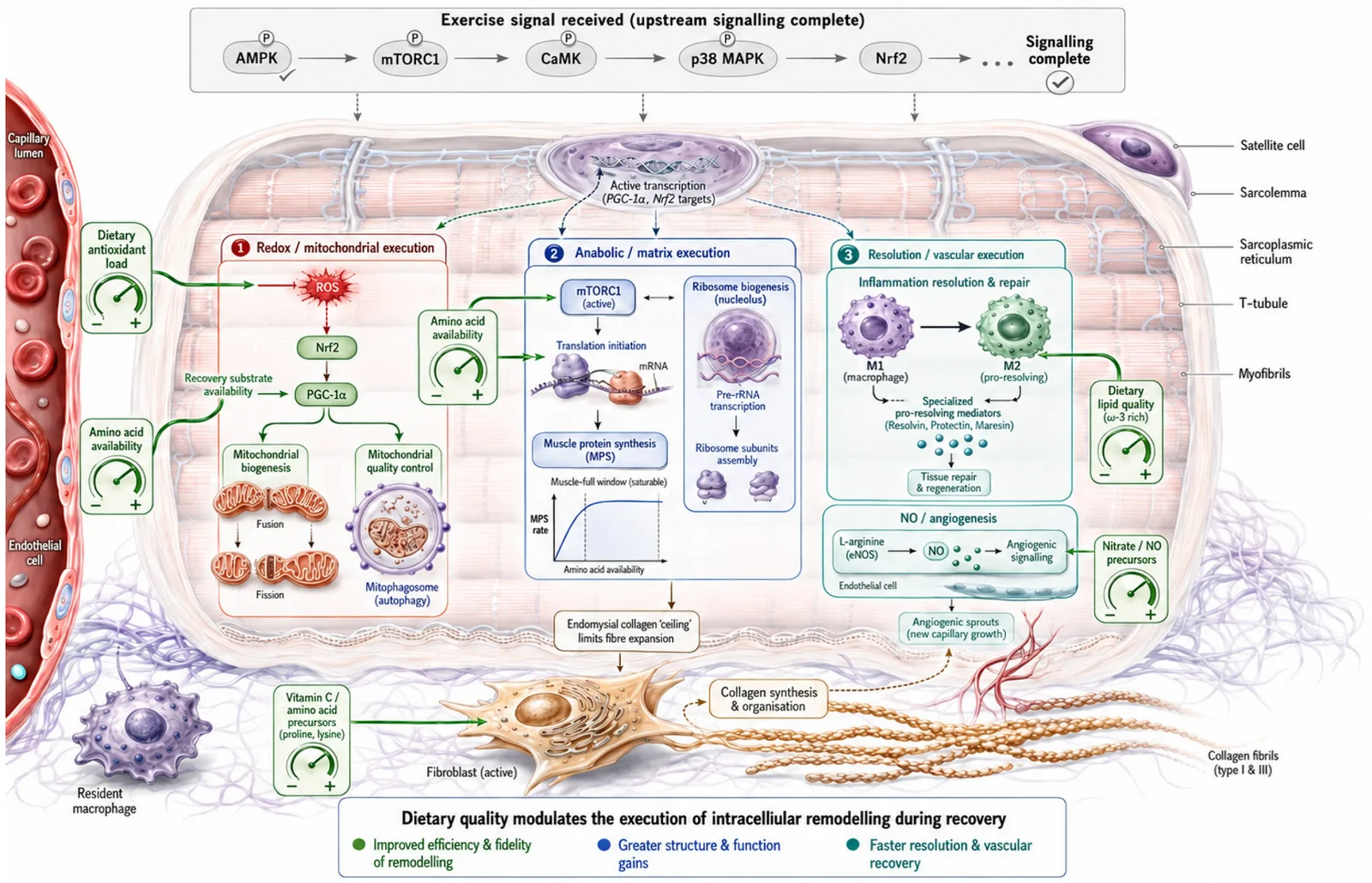
Nutritional modulation of the execution phase of exercise adaptation. Exercise-initiated signalling is followed by mitochondrial–redox, anabolic–matrix and immune–vascular remodelling during recovery. Nutritional conditions may provide substrates or modify regulatory environments relevant to these processes, but the relationships shown differ in evidential directness and should not be interpreted as established effects of habitual dietary quality on long-term adaptation. Exercise remains the indispensable initiating stimulus. Ellipses (…) indicate omitted intermediate molecular steps or processes for visual simplification. Solid coloured arrows denote the principal directional relationships within the depicted execution/remodelling pathways, whereas dashed coloured arrows indicate potential or context-dependent nutritional modulation of these processes rather than established causal effects. Arrow colours are used to distinguish functional modules and do not encode evidential strength, magnitude of effect or a favourable/unfavourable direction.

Dietary protein should not be interpreted through a single universal threshold for exercise adaptation. The European reference intake of approximately 0.83 g·kg^−1^·day^−1^ represents a population-level reference for protein requirements [50]. Meta-analytic evidence indicates that protein supplementation during resistance training can produce a small additional increase in fat-free mass (0.30 kg, 95% CI 0.09–0.52), with Morton et al. identifying a modelled breakpoint near 1.62 g·kg^−1^·day^−1^ beyond which additional supplementation was not associated with further gains [51]. This breakpoint should not be interpreted as a physiological requirement: the contributing trials evaluated supplementation across heterogeneous baseline diets, populations and training programmes rather than experimentally determining the minimum protein intake required for adaptation [46,51,52]. Accordingly, current supplementation evidence does not establish a physiological requirement above the population reference intake for exercise-induced remodelling. Within the present framework, the relevant boundary is whether protein availability is adequate rather than whether a universal intake target is exceeded. Once protein provision is not limiting, additional intake cannot be assumed to improve the execution of exercise-induced remodelling [28,46,51,52]. Execution also requires sufficient translational capacity and structural remodelling during recovery. Ribosome biogenesis expands the cellular machinery available for protein synthesis and may contribute to interindividual differences in hypertrophic adaptation [27,28]. Connective-tissue remodelling represents an additional structural component. Experimental connective-tissue models indicate that matrix synthesis responds to loading and recovery conditions [53], but current evidence does not establish the extracellular matrix as a rate-limiting constraint on human muscle-fibre hypertrophy. A small human mechanistic study reported increased collagen synthesis when vitamin C-enriched gelatin was provided before intermittent mechanical loading [54]. Whether this acute response translates into superior long-term connective-tissue adaptation remains uncertain. Proteostatic clearance through autophagic and related quality-control processes may also contribute to recovery remodelling [8,37], although the extent to which habitual diet modifies these processes sufficiently to alter human training adaptation remains uncertain. Mechanical loading initiates anabolic signalling, whereas nutritional conditions may influence how efficiently that signalling is translated into structural remodelling during recovery (Figure 3).

### 5.3. Immune Regulation and Endothelial Function

Biological adaptation extends beyond protein synthesis and includes the resolution of exercise-induced inflammatory responses [29,30]. Inflammatory resolution is an active process involving specialized pro-resolving mediators (SPMs), including lipoxins, resolvins, protectins and maresins [55]. Several SPM families derive from polyunsaturated fatty-acid precursors [55,56], providing a plausible route through which habitual fatty-acid intake could influence precursor availability. Whether altering dietary lipid quality meaningfully changes post-exercise inflammatory resolution or subsequent human training adaptation remains uncertain [55,56]. Vascular execution strictly follows a parallel biological structure. As established previously, physical exercise imposes continuous vascular shear stress that directly drives endothelial signalling and subsequent angiogenic remodelling [3]. Nitric oxide bioavailability contributes importantly to endothelial signalling and exercise-induced vascular adaptation. Diet can robustly support this bioavailability through a physiological route independent of the classical L-arginine pathway [57]. Dietary inorganic nitrate, highly abundant in specific leafy plants, undergoes systematic reduction through an enterosalivary and microbial pathway [11,57] to yield nitrite and ultimately NO [57]. This nitrate–nitrite–NO pathway can increase systemic NO availability [57]. It therefore provides a mechanistically plausible route linking dietary composition to vascular physiology, but evidence that habitual nitrate-rich dietary patterns enhance exercise-induced angiogenic adaptation remains limited [12,57].

Immune resolution and endothelial remodelling illustrate the systemic nature of the execution phase [30,55,56,57]. Their interaction is biologically plausible, but current evidence does not establish that manipulating dietary quality during recovery determines the structural quality of subsequent tissue adaptation. These pathways are therefore included as candidate execution mechanisms rather than as demonstrated diet-dependent determinants of training response.

### 5.4. Repeated Execution Across the Lifespan

Across the lifespan, repeated exercise responses occur against a changing biological background. Ageing is accompanied by alterations in endothelial function, inflammatory activity, nutrient sensing and other processes that overlap with domains represented in the ACS [31,32,44,58]. These changes may modify the context in which successive exercise bouts are processed, but it has not been established that habitual dietary quality preserves training responsiveness by directly modifying an ACS-specific execution mechanism. The lifespan relevance of the model should therefore be viewed as a testable extension of the framework rather than as an established consequence of nutritional modulation [31,32,44,58].

Once exercise has generated the initiating biological perturbation, nutritional conditions may influence several processes involved in its subsequent execution, including redox-sensitive transcription, substrate availability for anabolic and matrix remodelling, inflammatory resolution and endothelial function (Table 1). The available evidence varies substantially in directness, and these mechanisms should not be interpreted as demonstrating that habitual dietary quality determines the resulting adaptive phenotype.

## 6. The Adaptive Cellular State Model of Exercise–Diet Interaction

Physiological responses to exercise training exhibit substantial interindividual heterogeneity, with markedly different adaptive trajectories observed even after comparable training programmes [5]. Known individual characteristics contribute to this variability but do not fully account for it. The ACS Model addresses this unexplained variation by positioning a pre-existing multidimensional biological state between the prescribed exercise exposure and subsequent adaptation. ACS is therefore treated as a candidate moderator that must be characterized before the adaptive outcome it is intended to explain or predict, rather than inferred retrospectively from that outcome.

Habitual diet is proposed as a potentially important modifiable determinant of this adaptive context. Beyond influencing substrate availability and glycogen restoration, dietary exposures may shape mitochondrial and metabolic function [19,38], as well as vascular, inflammatory, microbial and epigenetic conditions relevant to the integration of repeated exercise signals [31,39,44]. A prescribed external workload does not, however, generate an invariant internal perturbation: substrate availability, hydration, vascular function and baseline metabolic status may alter the physiological strain experienced by the organism [19,38]. Exercise therefore remains the indispensable initiating perturbation [1,2,3]. Acute or proximal nutritional conditions may modify the internal perturbation generated by a prescribed workload, whereas habitual diet may shape the pre-existing and recovery context in which that perturbation is subsequently processed.

A central prediction of the model, summarized in Box 1, is that prespecified baseline ACS-related domains may provide information about subsequent training adaptation beyond conventional resting cardiometabolic and fitness measures. Individuals with similar conventional profiles could therefore differ in the biological configuration present before training and in their subsequent adaptive trajectories. The model does not prescribe a dietary identity: nutrient adequacy, overall dietary quality, food processing and the foods displaced by alternative choices are treated as the relevant exposure dimensions. Patterns rich in minimally processed plant foods represent one candidate configuration [12,17], supported by mechanistic evidence relevant to selected metabolic, microbial and vascular domains [39,57], but high-quality omnivorous patterns may share many of the same characteristics. Whether dietary quality provides incremental information about adaptation beyond energy, protein and conventional metabolic health remains an empirical question.

Box 1 **Definition, construct boundaries and falsifiable propositions of the Adaptive Cellular State Model.**

**Canonical definition.** The Adaptive Cellular State (ACS) is the pre-existing, dynamic and multidimensional biological configuration of cells and tissues that conditions how exercise-induced perturbations are received, decoded, executed and retained across repeated adaptive cycles.

**Canonical domains.** The model comprises nine interacting domains: mitochondrial quality, metabolic flexibility, nutrient sensing, redox responsiveness, inflammatory tone, microbiota-derived signalling, endothelial function, proteostasis and epigenetic readiness. These domains are not proposed as novel biological processes, validated ACS measurements or components that must vary in the same direction.

**Construct boundaries.** ACS differs from metabolic health because it is defined specifically in relation to subsequent adaptive responsiveness rather than resting cardiometabolic status alone. Metabolic flexibility represents one ACS domain rather than the construct itself. Trainability or responder status describes the adaptation that has occurred, whereas ACS must be characterized before that response. Hormetic preconditioning concerns altered responsiveness following previous stress exposure and is narrower than the proposed multidomain state. Allostatic load represents accumulated physiological burden rather than adaptation-specific competence. Short-term readiness reflects transient preparedness for an exercise bout, whereas ACS is intended to represent a more persistent biological context.

**A priori measurement rule.** Candidate ACS domains must be assessed before the training period and evaluated against independently measured subsequent adaptation. Retrospectively assigning a favourable ACS because an individual adapted well constitutes circular reasoning and is not a valid test of the model. No unvalidated aggregate ACS score should be constructed solely for this purpose.

**P1—Incremental validity.** Prespecified baseline ACS-related measures should provide reproducible information about subsequent adaptation beyond conventional cardiometabolic status, baseline fitness and training history. Failure to provide incremental information would materially weaken the construct.

**P2—Multidimensionality.** ACS-related domains need not vary concordantly within an individual. If they collapse into a single general-health axis without additional adaptation-specific information, the proposed multidimensional construct would be weakened.

**P3—Phenotype specificity.** The ACS domains most relevant to adaptation should differ according to the exercise modality and outcome. A single invariant profile predicting endurance, hypertrophic, vascular and metabolic adaptation equally well would argue against this proposition.

**P4—Dietary modulation.** Adequately controlled changes in habitual dietary quality should modify selected ACS-related domains independently of energy and protein provision if dietary quality contributes meaningfully to the model. Consistently null findings across well-controlled interventions would weaken its dietary component.

**P5—State-to-adaptation linkage.** Candidate ACS-related measures must prospectively relate to durable, independently assessed adaptation rather than only to acute pathway activation. Reproducible failure of baseline ACS-related measures to predict or track subsequent adaptation would require revision or rejection of the proposed framework.

### 6.1. Model Architecture

The ACS Model is a mechanistic heuristic rather than a validated causal or predictive model. It organizes six functionally connected components linking prescribed exercise exposure, the internally generated perturbation, the pre-existing ACS profile, execution and remodelling, the resulting adaptive phenotype and the biological state entering subsequent exercise bouts.

First, the prescribed exercise dose constitutes the external input, specified by modality, intensity, duration, frequency, volume and recovery structure [1,2,38].

Second, this workload generates the internal perturbation actually experienced by the organism. Energetic, mechanical, Ca^2+^-dependent and redox signals [1,2,3] interact with vascular, myokine and immune–endocrine responses [4,59]. Comparable prescribed workloads may nevertheless produce different internal stimuli because glycogen availability and substrate use [19,38], together with hydration, cardiorespiratory capacity and vascular or metabolic function [2,38], modify the resulting physiological strain.

Third, the internal perturbation occurs within a pre-existing multidimensional ACS profile comprising mitochondrial quality, metabolic flexibility, nutrient sensing, redox responsiveness, inflammatory tone, microbiota-derived signalling, endothelial function, proteostasis and epigenetic readiness [8,19,20,22,25,27,29,30,31,35,36,39,57]. These domains may differ in direction and relevance within the same individual and across adaptive phenotypes. Although skeletal muscle is central to many exercise responses, the relevant biological context also involves vascular, immune, stromal, hepatic, adipose and microbial compartments [2,4,29,39].

Fourth, the model proposes that this pre-existing configuration may condition how the internally generated perturbation is decoded and executed. Acute pathway activation must subsequently be translated into mitochondrial biogenesis and quality control [8,25,26], ribosome biogenesis and protein synthesis [27,28,46], and extracellular-matrix, vascular and immune remodelling [29,30,57]. Because pathway activation does not guarantee completed adaptation, the execution phase also depends on substrate availability and coordinated proteostatic, redox, inflammatory and nutrient-sensing processes [20,22,31,38].

Fifth, these processes produce the adaptive phenotype. Depending on the exercise modality and biological context, this may include greater mitochondrial respiratory capacity and metabolic flexibility [1,19,38], improved insulin sensitivity, endothelial function and capillarization [1,3,57], increased muscle mass and strength [27,28,46], or higher cardiorespiratory fitness and resistance to subsequent metabolic stress [1,5,38]. The contribution of individual ACS domains is therefore expected to vary across exercise modalities and across athletic, ageing and clinical populations [2,5,38].

Sixth, adaptation feeds back into the biological state entering subsequent exercise bouts. Prior exercise can produce persistent mitochondrial and translational remodelling, changes in tissue organization and epigenetic regulation [25,26,27,28,35]. ACS is therefore conceived as dynamic and history-dependent, with its configuration potentially changing with previous exercise, habitual diet, recovery, ageing and disease [31,34,35,39,44].

### 6.2. Emergent Properties and Dietary Modulation

Across this architecture, the model proposes four emergent functional properties. Gain denotes the extent to which an internally generated exercise signal produces molecular, structural and functional remodelling. Fidelity denotes whether energetic, mechanical, redox and inflammatory information is decoded in a coordinated manner appropriate to the stimulus. Biological noise denotes the chronic metabolic, inflammatory and oxidative background against which the transient exercise response occurs. Memory denotes the persistence of previous adaptive cycles and their influence on subsequent responsiveness. These properties are conceptual and have not yet been operationalized or validated as measurements; their purpose is to distinguish ACS from a generic description of cellular health and to generate directional, testable propositions.

**Figure 4:**
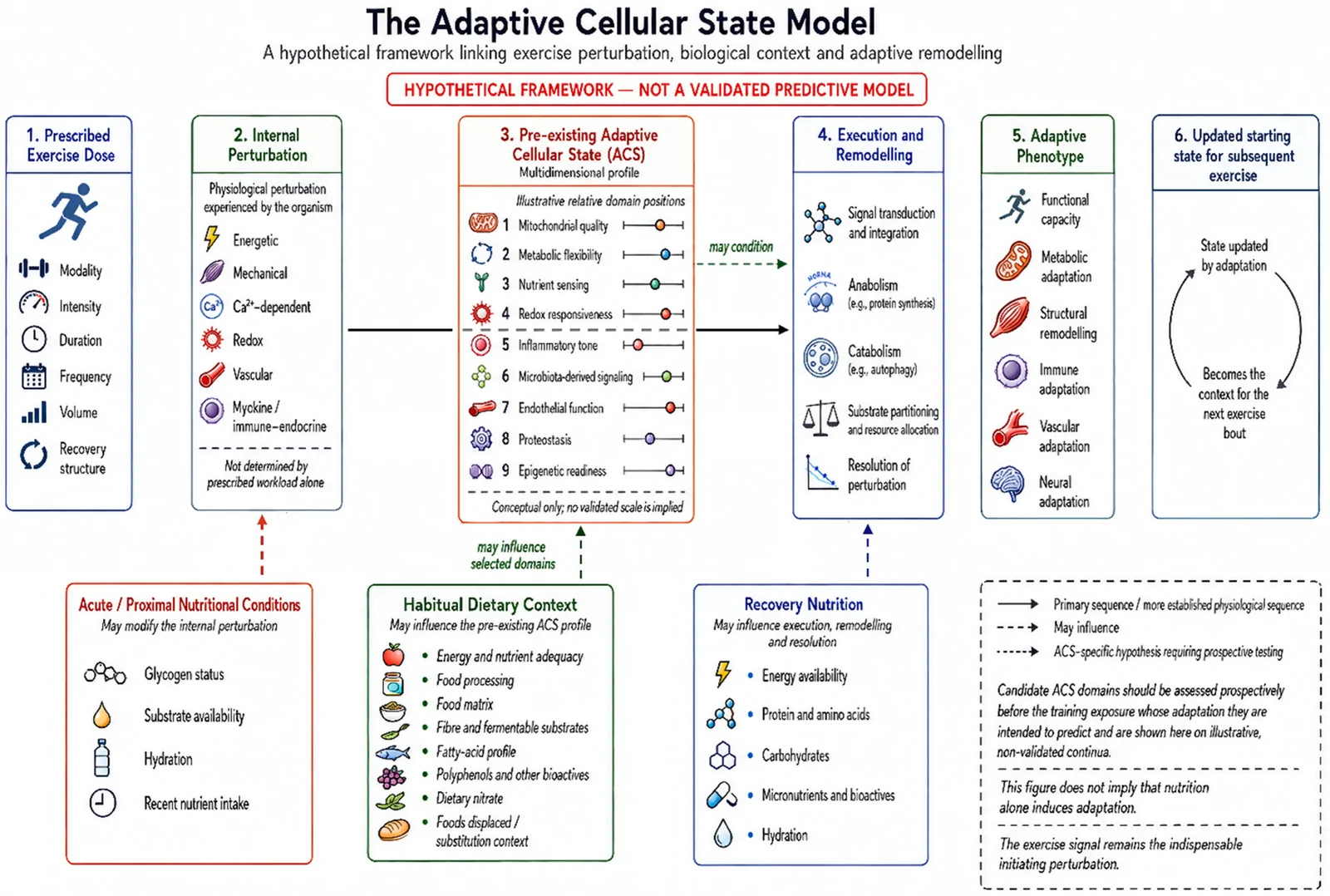
The Adaptive Cellular State (ACS) Model as a hypothetical framework for exercise–diet interaction. A prescribed exercise dose generates an internal perturbation that occurs within a pre-existing multidimensional ACS profile and is followed by execution, tissue remodelling and an adaptive phenotype. ACS comprises nine interacting domains, whereas gain, fidelity, biological noise and memory are proposed emergent properties rather than validated measurements. Habitual diet, acute nutritional conditions and recovery nutrition are shown as potentially distinct modifiers of different stages of the model. Arrows represent conceptual relationships of varying evidential directness rather than validated causal pathways. The framework does not define high- or low-ACS clinical categories and does not imply superiority of a specific dietary pattern.

Nutritional influences may occur at three distinct but overlapping stages. First, habitual diet may contribute to the pre-exercise context through longer-term effects on metabolic flexibility, inflammatory tone, microbial signalling and epigenetic regulation [19,31,39]. Second, acute or proximal nutritional conditions may modify the internal perturbation generated by a prescribed workload through glycogen and substrate availability, hydration, metabolic capacity and vascular function [19,38,57]. Third, nutritional provision during recovery supplies substrates and cofactors relevant to mitochondrial, proteostatic and vascular remodelling [38,46,57]. These routes should not be treated as a single dietary mechanism, and none reproduces the multidimensional contraction-derived exercise stimulus itself [1,2,3,4]. Figure 4 integrates these stages within the proposed architecture.

The model further proposes that adaptive regulation is non-linear. Physiological reactive oxygen species pulses support redox-sensitive adaptation, whereas high-dose antioxidant supplementation may attenuate selected training responses [22,23,24,47,48]. Acute mTORC1 activation supports anabolic remodelling, but chronically elevated nutrient signalling may reduce acute responsiveness [20]. Similarly, transient inflammation supports repair, whereas chronic metaflammation increases biological noise and disrupts resolution [29,30,31]. ACS should therefore not be represented as a uniformly favourable or unfavourable state. Rather, the model proposes that the relevance of individual domains depends on proportionality, timing, biological context and the adaptive outcome under consideration.

Together, these principles motivate the conditional dietary-quality hypothesis examined below. Under adequate energy, protein and essential nutrient provision, patterns emphasizing minimally processed foods and a diverse plant-food component are plausible candidates for influencing selected metabolic, inflammatory and microbial ACS-related domains [13,15,17,31,39]. This does not imply that such patterns produce a uniformly favourable ACS profile or greater adaptation than appropriately constructed high-quality omnivorous comparators.

### 6.3. A Conditional Nutritional Hypothesis: Dietary Quality and Plant-Rich Patterns

The ACS Model generates a conditional dietary-quality hypothesis: provided that total energy, protein and essential nutrient requirements are met, dietary patterns containing a greater proportion and diversity of minimally processed plant foods may influence selected ACS-related domains [12,13,17]. This proposition concerns a continuum of dietary quality rather than vegan, vegetarian or omnivorous identity. Food processing, nutrient density, food matrix, protein adequacy, adherence and the foods displaced by alternative choices may be more informative than the inclusion or exclusion of animal-source foods alone [14,15,17].

Mechanistic plausibility arises from several co-occurring dietary characteristics. Fibre and fermentable substrates modify microbial ecology and short-chain fatty-acid production [9,10,11,39]; polyphenols and their microbial metabolites may engage redox, inflammatory and mitochondrial quality-control pathways [22,42,43,60]; unsaturated fatty acids provide substrates relevant to inflammatory resolution [55,56]; and dietary nitrate may increase nitric-oxide availability and endothelial signalling [57]. These pathways map onto domains represented in the ACS Model, but their coexistence within a dietary pattern does not itself demonstrate enhanced exercise adaptation.

Within the model, lower chronic inflammatory activity may reduce biological noise [29,30,31], whereas greater insulin sensitivity and metabolic flexibility may support the coordination of nutrient-sensing processes during exercise and recovery [19,38]. Preserved endothelial function may facilitate perfusion, substrate delivery and vascular remodelling [57,58], while microbiota-derived metabolites may influence mitochondrial, immune and epigenetic regulation [39,60,61]. These pathways therefore generate testable hypotheses concerning gain, fidelity, biological noise and memory, but current evidence does not establish that a habitual whole-diet pattern modifies these properties in humans.

The relevant comparator must also be specified. A high-quality omnivorous pattern containing abundant whole plant foods, fish and other minimally processed foods may share many biological characteristics with a well-planned vegetarian pattern. Conversely, a vegan diet dominated by refined or ultra-processed products may not provide the proposed advantage [14,15,17]. Comparisons should therefore be substitution-based—for example, legumes replacing processed meat, nuts replacing refined snacks, or whole grains replacing refined grains—because increasing one dietary component necessarily displaces another or changes total energy intake [12,17]. The hypothesis concerns dietary composition, quality and displacement rather than dietary labels alone.

This hypothesis has important nutritional and phenotype-specific boundaries. Energy deficiency can impair training quality, endocrine function and recovery [20,38], whereas inadequate protein intake, digestibility, indispensable amino-acid provision or distribution may constrain muscle protein synthesis and hypertrophic adaptation [46,49,50,51,52]. Moreover, high-fibre, lower-energy-density dietary patterns may make energy sufficiency more difficult to maintain in individuals with high energy expenditure or limited appetite; a plant-rich pattern should therefore not be expected to support adaptation if total energy or nutrient adequacy is compromised [14,17,38]. Vegan diets additionally require attention to vitamin B12 and, depending on food selection and supplementation, iodine, iron, zinc, calcium, vitamin D and long-chain omega-3 fatty acids [17]. No whole-diet superiority has been established across hypertrophic, endurance, vascular or clinical adaptive phenotypes, and the direction and magnitude of any dietary modulation may vary by exercise modality, outcome, population and baseline biological context.

Direct human evidence remains limited. Dietary-pattern studies indicate that plant-rich patterns can modify several cardiometabolic and biological systems represented in the ACS [12,13,15,17], but this does not demonstrate greater long-term exercise adaptation under standardized training and appropriately controlled dietary conditions. Nutrient-adequate, minimally processed plant-rich patterns should therefore be considered candidate exposures for testing selected ACS-related domains, not proven means of improving ACS or universally amplifying training responses.

### 6.4. Critical Tests and Unresolved Questions

The ACS Model raises several questions that must be resolved before it can progress from a mechanistic framework to an operational model. First, dietary quality is a composite exposure. Food matrix, fibre content, protein source, fatty-acid profile, degree of processing and the foods displaced by a given substitution covary within real-world dietary patterns [12,17,60,61]. Observational associations therefore cannot readily determine which components are causal or whether their effects are additive, redundant or mediated through shared biological pathways [62,63]. The model should consequently be tested using explicitly defined dietary contrasts and substitutions rather than broad dietary labels alone.

Second, total and composition-specific dietary effects must be distinguished. A dietary pattern may influence training adaptation indirectly through changes in energy intake, adiposity, insulin sensitivity, glycogen availability or protein adequacy [14,19,38,46,51]. An ad libitum dietary-pattern trial therefore estimates a different effect from an energy- and protein-matched trial: the former captures the total effect of adopting the pattern, whereas the latter asks whether dietary composition contributes beyond energy and protein provision. Both questions are relevant, but they require different interpretations, and no single trial would validate or refute the complete ACS Model.

Third, several proposed relationships are likely to be non-linear. Exercise-induced reactive oxygen species contribute to redox-sensitive mitochondrial and antioxidant adaptation [22,23,24], whereas high-dose vitamin C or E supplementation may attenuate selected molecular and training responses [47,48]. The relevant boundary probably depends on dose, timing, chemical form, baseline redox status and whether compounds are delivered as isolated supplements or within a food matrix. The testable proposition is therefore not that greater antioxidant exposure is uniformly beneficial, but that adaptation depends on proportional redox activation followed by appropriate resolution.

Fourth, dietary modulation is unlikely to be uniform across individuals, tissues or adaptive phenotypes. Postprandial and glycemic responses vary according to baseline clinical and microbiome characteristics [64,65], while exercise-induced molecular responses differ across metabolic contexts [5,66]. Whether dietary modulation of ACS-related processes is greater in older adults or in individuals with obesity, insulin resistance or chronic inflammation than in trained, metabolically healthy individuals remains an effect-modification hypothesis rather than an established conclusion. It should be tested across prespecified populations, exercise modalities, tissues and adaptive outcomes.

Finally, ACS remains an unvalidated latent construct. No single biomarker captures all its proposed domains, and combining multiple measures into an arbitrary score would introduce problems of weighting, overfitting and reproducibility. Candidate ACS domains should therefore be prespecified and measured before the training exposure whose adaptation they are intended to predict. Until a composite measure is prospectively developed and externally validated, studies should use domain-based panels rather than an undifferentiated ACS score. A provisional research panel could include insulin sensitivity or metabolic flexibility [19], mitochondrial respiratory function [25,67,68], endothelial function or nitrate–nitric oxide signalling [57], basal inflammatory activity [31], and indicators of proteostatic or remodelling capacity [8,46]. Multi-omic and single-cell approaches may help derive candidate signatures [67,68,69,70], but these must demonstrate reproducibility, temporal responsiveness and incremental predictive value for durable adaptation beyond conventional cardiometabolic markers before they can be interpreted as measures of ACS.

These questions define discriminating tests of the framework rather than a research programme designed only to confirm it. Evidence that dietary-pattern quality contributes no information beyond energy, protein and conventional metabolic-health indicators, or that candidate ACS measures do not relate prospectively to durable adaptation, would materially weaken the model. Table 2 summarizes the principal questions, informative study designs, core measurements and threats to inference.

### 6.5. Evidence That Constrains the Hypothesis

Available human evidence also places important constraints on the dietary component of the model. In the controlled-crossover OMNIVEG study, transition from a traditional to a vegan Mediterranean dietary pattern did not produce a clearly detectable change in peak fat oxidation (0.323 ± 0.153 vs. 0.347 ± 0.147 g·min^−1^; *p* = 0.678), Fatmax (40.51 ± 7.30 vs. 40.51 ± 10.71% VO_2_peak; *p* = 1.000), or the exercise-intensity response curves for fat oxidation, heart rate or perceived exertion [71]. This directly relevant null finding does not validate or refute the ACS Model, because acute substrate utilization is not equivalent to durable training adaptation. It does, however, demonstrate that a substantial change in dietary pattern need not produce a detectable change in every exercise-related phenotype.

Other evidence similarly cautions against a simple direction-of-benefit interpretation. High-dose antioxidant interventions show that nutritional exposures capable of modifying redox biology may attenuate rather than enhance selected exercise responses [47,48]. Together, these observations reinforce two boundaries of the framework: ACS domains may change discordantly, and mechanistically plausible dietary exposures need not improve all adaptive outcomes. Evidence that prospectively measured ACS-related domains consistently fail to add predictive information beyond conventional markers, or that well-controlled changes in dietary quality fail to modify the proposed domains or subsequent adaptation, would provide a more direct challenge to the model.

## 7. Limitations, Future Research and Clinical Translation

### 7.1. Limitations and Current Claim Boundary

Interpretation of the ACS Model should be framed by the nature and maturity of the evidence on which it is built. Much of the supporting literature derives from cellular or animal models and from interventions using isolated compounds [23,39,41,42,43,45]. Such studies can establish pathway engagement and mechanistic plausibility, but they cannot demonstrate integrated human remodelling or improved long-term training adaptation. Direct diet–training evidence remains sparse and is concentrated in isolated nutritional exposures, acute physiological outcomes or relatively short-term interventions [47,48,71]. Mechanistic coherence should therefore not be interpreted as evidence of whole-diet efficacy. The evidence synthesis also has review-level limitations. This work was conducted as a critical narrative review rather than a systematic review; literature identification and evidential weighting were purposive and partly judgement-dependent. No exhaustive retrieval, duplicate study selection, formal risk-of-bias assessment or certainty grading was undertaken. Although null and contradictory evidence was deliberately considered, selective emphasis and confirmation bias cannot be excluded. The model should therefore be judged primarily through prospective tests of its propositions rather than through the apparent coherence of the literature assembled here.

Dietary exposure is inherently heterogeneous. Vegan, vegetarian, plant-rich and omnivorous labels can encompass substantial differences in food processing, nutrient density, fibre, fatty-acid composition, protein quality and micronutrient adequacy [14,15,17]. A minimally processed omnivorous pattern may therefore share more biological characteristics with a high-quality vegetarian pattern than with an ultra-processed diet [17]. Because dietary changes also involve displacement, observed effects may depend on what is replaced as much as on what is added [12,17]. Dietary identity alone is therefore an insufficient causal exposure for testing the ACS hypothesis.

Energy balance, body composition and protein provision create a related causal problem. Low energy availability can impair training and recovery [38], while energy intake, adiposity and nutrient sensing may themselves change after dietary modification [14,20]. Protein availability can also constrain selected adaptive outcomes [46,49,50,51,52]. These variables may be components or mediators of the dietary intervention rather than conventional baseline confounders. Consequently, an ad libitum dietary-pattern trial estimates a different effect from an energy- and protein-matched trial, and post-randomization adjustment for these pathways is not equivalent to controlling them by design.

Much of the long-term dietary-pattern evidence relevant to cardiometabolic, inflammatory and vascular physiology is observational [61,72,73,74,75,76] and therefore remains vulnerable to residual confounding, healthy-user bias, reverse causation and dietary measurement error [62,63]. Such evidence can support external plausibility but cannot establish that dietary quality causally modifies exercise adaptation or the ACS-related pathways proposed here.

Outcome selection and timing also constrain interpretation. Insulin sensitivity, metabolic flexibility, inflammatory activity, endothelial function and substrate oxidation may represent ACS-related domains [19,31,38,57,71], but they are not interchangeable with durable mitochondrial remodelling, hypertrophy, cardiorespiratory fitness or functional adaptation. Acute pathway activation likewise does not establish phenotype consolidation [48,67,68]. Studies must therefore distinguish baseline state, internal perturbation, recovery responses, tissue remodelling and longer-term functional outcomes.

Dietary modulation is unlikely to be uniform across adaptive outcomes or populations. The relevance of individual ACS domains may differ between mitochondrial, vascular, metabolic and hypertrophic phenotypes, the latter being particularly dependent on adequate energy and protein provision [19,31,38,46,49,50,51,52,57]. Age, sex, baseline fitness, training history, metabolic health and microbiome characteristics may further contribute to heterogeneity [5,64,65,66]. Whether any dietary effect is larger in older adults or in individuals with obesity, insulin resistance or chronic inflammation remains an effect-modification hypothesis rather than an established conclusion.

ACS remains an unvalidated latent construct. No single biomarker captures its nine proposed domains, and an arbitrary aggregate score would introduce problems of weighting, overfitting and reproducibility. Whole-tissue measurements may also obscure opposing or cell-specific responses [67,68,69]. Multi-omic, single-cell and spatial approaches may help derive candidate signatures [67,68,69,70], but these must be measured prospectively and validated against independent, durable adaptation before they can be interpreted as ACS-related measures. The model must likewise remain biologically decomposable across muscle, vascular, immune, metabolic and microbial compartments [2,59,67,68].

These limitations define the current claim boundary. Dietary quality, including nutrient adequacy, processing, food matrix and displacement, is proposed as one potential modifier of selected ACS-related domains [12,13,17], but current evidence does not establish that any dietary label produces a superior ACS profile or greater training adaptation. The ACS Model should therefore be treated as a heuristic for generating discriminating, prospective experiments rather than as a validated predictive measure or clinical prescription. Its central propositions require direct diet–exercise testing, longitudinal molecular and functional outcomes and external validation of candidate ACS-related measures.

### 7.2. Future Research and Clinical Translation

These limitations define a staged research agenda for testing and operationalizing the ACS Model before clinical translation is considered (Table 3). Future trials should distinguish two related but different causal questions. Energy- and protein-matched feeding trials can estimate composition-specific dietary effects under controlled nutritional conditions, whereas pragmatic dietary-pattern trials can estimate the total effect of adopting a dietary pattern, including pathways operating through energy intake, adiposity, food processing and metabolic health. Factorial designs crossing exercise programmes with contrasting dietary patterns would be particularly informative. Where long-term randomization is infeasible, target-trial emulation in well-characterized cohorts may strengthen causal inference, provided that eligibility, treatment strategies, time zero, follow-up, outcomes, confounders and the target estimand are explicitly defined [77].

Studies should also characterize adaptation as a trajectory rather than rely on isolated post-exercise snapshots. Longitudinal interventions with repeated molecular and functional measurements could distinguish the pre-exercise state, internally generated perturbation, recovery response and durable adaptive phenotype [67,68]. Single-cell and spatial approaches may help localize responses to specific cellular populations, but they are not required for every test of the model. Stable-isotope flux analysis, high-resolution respirometry and validated functional outcomes remain important for linking molecular changes to measurable metabolic and physiological behaviour.

Multi-omic integration and appropriately constrained statistical-learning approaches may assist in deriving candidate ACS-related signatures [64,70,78]. Model development should use adequate sample sizes, prespecified predictors, appropriate shrinkage or penalization, internal validation and subsequent external validation. Candidate measures must be obtained before the adaptation they are intended to predict and demonstrate reproducibility, calibration and incremental predictive value beyond conventional variables such as age, sex, disease status, baseline fitness, training history and resting cardiometabolic markers. Association with acute molecular responses alone would be insufficient.

Clinical translation should remain secondary to construct validation. If prospectively and externally validated, ACS-related assessment might eventually support treatment stratification or monitoring rather than disease diagnosis, particularly where exercise is therapeutic and response heterogeneity has meaningful consequences. Before such use is justified, candidate measures must add reproducible information beyond conventional clinical and fitness assessment and ultimately demonstrate that ACS-guided decisions improve patient-important outcomes. Training history, sleep quality, recovery status and psychological stress should also be prespecified as important non-dietary contextual modifiers rather than attributed to diet or absorbed into an undifferentiated ACS score. Older adults and individuals with obesity, insulin resistance or chronic cardiometabolic conditions may provide informative populations for testing effect modification [5,64,65,66], but greater benefit should not be assumed. Ultimately, an impact trial comparing ACS-guided assessment with conventional management would be required to establish clinical actionability.

## 8. Conclusions

Exercise provides the indispensable initiating perturbation for adaptation, whereas the biological context in which that perturbation is generated and subsequently processed may contribute to interindividual variation in training responses. The Adaptive Cellular State (ACS) is proposed as a pre-existing, dynamic and multidimensional biological configuration that may condition how exercise-induced perturbations are received, decoded, executed and retained across repeated adaptive cycles. Its nine domains and four emergent properties are theoretical components of a heuristic framework rather than validated measurements or clinical categories.

Habitual dietary quality is one potential modifier of selected ACS-related domains, but current evidence does not establish that any dietary pattern produces a superior ACS profile or greater exercise adaptation. The principal contribution of the ACS Model is therefore to organize established biological processes into prospective, discriminating and falsifiable hypotheses about exercise responsiveness. Its validity will depend on whether prespecified baseline ACS-related measures predict durable adaptation beyond conventional cardiometabolic, fitness and training-history variables, and whether experimental evidence supports, refines or ultimately refutes the proposed relationships.

## Figures and Tables

**Table 1:** Candidate nutritional influences on processes involved in the execution of exercise-induced adaptation. The table distinguishes mechanistic plausibility from demonstrated effects on adaptive outcomes and summarizes the predominant level of evidence and principal remaining uncertainties.

Cellular Process	Principal Molecular Regulators	Potential Nutritional Relevance	Predominant Level of Evidence	Remaining Uncertainties
Mitochondrial biogenesis and quality control	PGC-1α, AMPK, p38 MAPK, mitophagy (ULK1)	Recovery substrate and redox context permit or constrain transcription and sustainment of the biogenic programme [25,26,38,47,48]	Human mechanistic; animal	Whether food-level (vs. high-dose supplemental) antioxidant intake meaningfully alters biogenesis; dose–timing thresholds
Redox signalling and ROS hormesis	ROS, Nrf2, antioxidant-response genes	High-dose antioxidant intake around training can blunt redox-dependent adaptation; ordinary dietary antioxidant quality uncertain [22,23,24,47,48]	Human intervention (endurance); animal	Form/dose specificity; frequent discordance between molecular signalling and performance outcomes
Metabolic flexibility and substrate switching	AMPK, PDK4, insulin signalling, lipid intermediates	Recovery energy/substrate availability shapes the metabolic milieu of adaptation [19,38]	Human mechanistic	Long-term adaptive (vs. acute signalling) consequences; interindividual variability
Muscle protein synthesis and anabolic execution	mTORC1, p70S6K, essential amino acids	Adequate amino acid provision supports MPS, but additional protein cannot be assumed to increase adaptation once protein availability is non-limiting [46,49,51,52].	Meta-analysis; human mechanistic	Minimum intake required to avoid protein limitation across populations and training contexts; source and distribution effects; no validated universal adaptive threshold.
Ribosome biogenesis (translational capacity)	rDNA transcription, c-Myc, mTORC1	Nutrient/energy-sensing tone supports expansion of translational capacity during recovery [27,28]	Animal; human mechanistic	Direct dietary-quality effects in humans largely untested
Extracellular matrix and connective-tissue remodelling	Fibroblast collagen synthesis, prolyl/lysyl hydroxylation (vitamin C cofactor)	Loading and recovery conditions influence connective-tissue remodelling; vitamin C-enriched gelatin has increased acute collagen synthesis in a small human mechanistic study [53,54].	Experimental connective-tissue models; small human mechanistic study	Translation to long-term tendon/matrix adaptation; limited and mixed clinical evidence
Autophagy and proteostasis	ULK1, AMPK, autophagy-supporting dietary inputs	Nutrient-sensing tone and specific inputs modulate the clearance that precedes anabolic renewal [8,37,45]	Animal; early human	Human dose–response; interaction with anabolic timing
Immune resolution	Specialized pro-resolving mediators (SPMs) from PUFA substrate	Fatty-acid availability provides precursors for specialized pro-resolving mediators; effects on post-exercise adaptation remain uncertain [55,56].	Animal; human mechanistic	Whether it enhances adaptation; risk of over-suppressing adaptive inflammation
Endothelial function and angiogenesis	NO (eNOS and nitrate–nitrite–NO pathway), VEGF, shear stress	Dietary nitrate can increase NO availability; whether this modifies training-induced vascular remodelling remains uncertain [57,58].	Human mechanistic; animal	Magnitude within training adaptation; dependence on oral/gut microbiota
Long-term adaptive capacity and vascular/healthy ageing	AMPK–SIRT–mTOR, inflammatory and oxidative pathways	Ageing-related inflammatory, metabolic and vascular changes overlap with ACS-related domains and may modify the context of repeated adaptation [31,32,37,44,58].	Human observational; animal	Causal contribution of diet–exercise interaction to healthspan; few long-term controlled data

**Table 2:** Discriminating tests of the Adaptive Cellular State Model. Each row links a falsifiable question to an informative study design, core measurements and the principal threat to inference. The table defines a research agenda rather than summarizing established effects. RCT, randomized controlled trial; RET, resistance exercise training; SPM, specialized pro-resolving mediator; MPS, muscle protein synthesis; SCFA, short-chain fatty acid; ACS, Adaptive Cellular State.

Research Question	Biological Domain	Ideal Study Design	Key Measurements	Main Methodological Risk
Do baseline ACS-related domains predict durable training adaptation beyond conventional cardiometabolic and fitness measures?	Construct and discriminant validity	Prospective training study or RCT with prespecified baseline ACS-domain assessment and longitudinal outcomes	Baseline ACS-domain panel; conventional cardiometabolic and fitness markers; independently assessed durable adaptation	Circular outcome-derived ACS definition; overfitting; post hoc predictor selection; absence of external validation
Does higher dietary pattern quality augment training-induced mitochondrial biogenesis and network quality independently of energy balance?	Mitochondrial adaptation	Energy- and protein-matched parallel RCT varying pattern quality during a standardized training block	Respiratory function (high-resolution respirometry), mtDNA copy number, PGC-1α/OXPHOS proteins, mitophagy markers	Confounding by adiposity and energy availability; muscle biopsy timing relative to bouts
Where is the dose–timing boundary between adaptive redox support and maladaptive ROS suppression from dietary antioxidants?	Redox signalling	Dose-ranging RCT of food-matrix vs. isolated antioxidants around training	Nrf2 target genes, redox biomarkers, mitochondrial adaptation, performance	Assuming monotonic effects; supplement dose not representative of dietary intake
Do fibre- and polyphenol-derived microbial metabolites causally enhance exercise adaptation?	Gut microbiota	Fibre/polyphenol intervention or defined-metabolite RCT with training; microbiota transfer in animal models for causality	SCFA and polyphenol-catabolite flux, metagenomics, host mitochondrial/immune readouts	Interindividual microbiome variability; correlation mistaken for causation
Does lowering chronic inflammatory tone through diet improve resolution of exercise inflammation and repair?	Immunometabolism	RCT in higher-inflammation populations with training; recovery time-course sampling	SPM/lipid mediators, cytokine kinetics, macrophage phenotype, satellite-cell activation	Over-suppressing adaptive inflammatory signalling; assay sensitivity for SPMs
Does dietary nitrate/plant-matrix intake enhance exercise-induced endothelial and angiogenic adaptation?	Endothelial function	Controlled-feeding RCT with endurance training; oral-microbiome stratification	NO bioavailability, flow-mediated dilation, capillarization, VEGF signalling	Oral/gut microbiota heterogeneity; ergogenic vs. adaptive effects conflated
Does dietary quality raise proteostatic/autophagic capacity to support post-exercise remodelling?	Proteostasis	Mechanistic human trial with tracer methods and autophagy-supporting dietary inputs	Autophagic flux, MPS kinetics, ubiquitin–proteasome markers	Measuring autophagy in humans; anabolic–catabolic timing interactions
Within plant-rich patterns, is protein adequacy sufficient to sustain hypertrophic adaptation?	Muscle remodelling	RCT comparing protein source/distribution within a matched plant-rich pattern plus RET	MPS, fractional synthetic rate, lean mass, strength	Protein quantity/quality confound; muscle-full ceiling; adherence
Is dietary tuning of adaptation larger where baseline ACS is more displaced (ageing, obesity, insulin resistance)?	Ageing/cardiometabolic disease	Stratified RCTs across age and metabolic-health strata with identical training	Composite ACS panel, insulin sensitivity, inflammatory tone, functional capacity	Effect-modification underpowered; generalizing across populations
Do dietary-quality effects differ between athletes and clinical populations in kind or only degree?	Athletic performance/cardiometabolic disease	Parallel trials in trained and clinical cohorts using harmonized protocols	Performance, molecular adaptation, ACS signature	Ceiling effects in athletes; non-comparable outcomes across cohorts
Can an integrated multi-omic signature define and track the adaptive cellular state?	Cross-domain (systems)	Longitudinal multi-omic profiling across tissues during diet × training	Transcriptomics, proteomics, metabolomics, epigenomics, microbiome, single-cell	Overfitting; signature not causally validated against outcomes

**Table 3:** Future research priorities for testing, operationalizing and, where justified, translating the Adaptive Cellular State Model. Proposed clinical applications remain hypothetical and require prospective validation and impact evaluation. Abbreviations: ACS: adaptive cellular state; FAP: fibro-adipogenic progenitor; OXPHOS, oxidative phosphorylation; RCT: randomized controlled trial; SCFA, short-chain fatty acid.

Research Priority	Knowledge Gap	Recommended Study Design	Key Biomarkers	Potential Impact
Isolate dietary quality from energy balance	Quality effects entangled with energy intake, adiposity and protein sufficiency	Factorial exercise × dietary-pattern RCT with energy and protein matched	Mitochondrial respiration, insulin sensitivity, PGC-1α/OXPHOS, inflammatory tone	Tests whether pattern quality contributes to adaptation independently of energy and protein provision
Test the energy-availability boundary	High-fibre or lower-energy-density patterns may compromise energy adequacy in individuals with high requirements or limited appetite	Controlled or pragmatic diet × training trial with prospective monitoring of energy intake and training exposure	Energy intake, body mass, training quality, recovery and relevant functional outcomes	Defines whether potential dietary-quality effects are constrained or reversed when energy adequacy is not maintained
Capture adaptation as a trajectory	Reliance on single-timepoint snapshots and surrogate endpoints	Longitudinal intervention with serial biopsies and repeated multi-omics	Time-resolved transcriptome, proteome, metabolome; functional capacity	Distinguishes transient signalling from durable remodelling
Emulate unfeasible long-term trials	Phenotype consolidation exceeds practical RCT duration	Target-trial emulation in well-characterized cohorts	Diet-quality indices, incident cardiometabolic and functional outcomes	Strengthens causal inference where randomization is impractical
Resolve cell-type-specific responses	Whole-tissue assays average away distinct cell populations	Single-cell and spatial transcriptomics of muscle and its niche	Cell-type transcriptomes; satellite-cell, FAP, endothelial, immune states	Localizes the adaptive cellular state to specific responding cells
Link molecular state to metabolic function	Expression data uncoupled from substrate handling	Stable-isotope flux analysis and mitochondrial phenotyping	Substrate oxidation fluxes, respirometry, metabolite tracing	Connects the model’s constructs to measurable metabolic behaviour
Define microbial-metabolite causality	Microbiome–host associations rarely shown to be causal	Metabolite or fibre/polyphenol intervention; microbiota transfer in models	SCFA and polyphenol-catabolite flux, metagenomics, host readouts	Tests whether diet-derived metabolites causally tune adaptation
Characterize redox dose–response	Boundary between adaptive support and maladaptive suppression undefined	Dose-ranging RCT of food-matrix vs. isolated antioxidants around training	Nrf2 targets, redox biomarkers, mitochondrial adaptation	Defines when antioxidant intake helps versus harms adaptation
Derive reproducible cross-domain ACS-related signatures	Parallel omic layers hard to fuse into a causal readout	Multi-omic integration with network/systems analysis	Cross-omic modules; network connectivity metrics	Identifies candidate cross-domain signatures without assuming a validated global ACS score
Operationalize the adaptive cellular state	No validated, direct biomarker of the construct	Prospective domain-based measurement followed by independent and external validation	Prespecified domain-based panel spanning mitochondrial, metabolic, redox, inflammatory, endothelial and other relevant ACS domains	Determines whether ACS-related domains can be measured reproducibly and predict subsequent adaptation
Model interindividual heterogeneity	Whether baseline ACS-related domains explain reproducible variation in dietary and training responses	Prediction studies integrating microbiome, metabolomics and clinical data	Personalized response predictors; postprandial and training responses	Tests incremental prediction before any individualized prescription is considered
Test effect modification by population	Unknown whether dietary or exercise effects differ according to baseline ACS-related profiles	Stratified RCTs across age, sex and metabolic-health strata	ACS panel, insulin sensitivity, inflammatory tone, functional capacity	Determines whether prespecified population or baseline-state interactions are reproducible
Evaluate clinical utility of ACS-guided assessment	Whether ACS adds actionable information beyond conventional assessment	External validation followed by an ACS-guided impact trial	Validated ACS-related measures; functional and patient-reported outcomes	Determines whether ACS improves decisions and outcomes

## Data Availability

No new data were created or analyzed in this study. Data sharing is not applicable to this article.

## Abbreviations

The following abbreviations are used in this manuscript:

ACS	Adaptive Cellular State
AKT	Protein kinase B
AMPK	5′ adenosine monophosphate-activated protein kinase
ATP	Adenosine triphosphate
BCAA	Branched-chain amino acids
CaMK	Calcium/calmodulin-dependent protein kinase
DNA	Deoxyribonucleic acid
eNOS	Endothelial nitric oxide synthase
FOXO	Forkhead box O transcription factors
HDAC	Histone deacetylase
LDL-C	Low-density lipoprotein cholesterol
MAPK	Mitogen-activated protein kinase
MPS	Muscle protein synthesis
mTOR	Mechanistic target of rapamycin
mTORC1	Mechanistic target of rapamycin complex 1
NAD^+^	Nicotinamide adenine dinucleotide
NF-κB	Nuclear factor kappa B
NO	Nitric oxide
Nrf2	Nuclear factor erythroid 2-related factor 2
PDK4	Pyruvate dehydrogenase kinase 4
PGC-1α	Peroxisome proliferator-activated receptor gamma coactivator 1-alpha
PUFA	Polyunsaturated fatty acids
RNA	Ribonucleic acid
ROS	Reactive oxygen species
SCFAs	Short-chain fatty acids
SIRT1	Sirtuin 1
SPMs	Specialized pro-resolving mediators
TMAO	Trimethylamine N-oxide
ULK1	Unc-51-like kinase 1
VEGF	Vascular endothelial growth factor
